# SENP7 senses oxidative stress to sustain metabolic fitness and antitumor functions of CD8^+^ T cells

**DOI:** 10.1172/JCI155224

**Published:** 2022-04-01

**Authors:** Zhongqiu Wu, Haiyan Huang, Qiaoqiao Han, Zhilin Hu, Xiao-Lu Teng, Rui Ding, Youqiong Ye, Xiaoyan Yu, Ren Zhao, Zhengting Wang, Qiang Zou

**Affiliations:** 1 Shanghai Institute of Immunology, Department of Immunology and Microbiology, State Key Laboratory of Oncogenes and Related Genes, Hongqiao International Institute of Medicine, Tongren Hospital, Shanghai, China.; 2 Department of General Surgery, Ruijin Hospital, Shanghai, China.; 3 Department of Gastroenterology, Ruijin Hospital, Shanghai Jiao Tong University School of Medicine, Shanghai, China.

**Keywords:** Immunology, Metabolism, Adaptive immunity, Cancer immunotherapy, T cells

## Abstract

The functional integrity of CD8^+^ T cells is tightly coupled to metabolic reprogramming, but how oxidative stress directs CD8^+^ T cell metabolic fitness in the tumor microenvironment (TME) remains elusive. Here, we report that SUMO-specific protease 7 (SENP7) senses oxidative stress to maintain the CD8^+^ T cell metabolic state and antitumor functions. SENP7-deficient CD8^+^ T cells exhibited decreased glycolysis and oxidative phosphorylation, resulting in attenuated proliferation in vitro and dampened antitumor functions in vivo. Mechanistically, CD8^+^ T cell–derived ROS triggered cytosolic SENP7–mediated PTEN deSUMOylation, thereby promoting PTEN degradation and preventing PTEN-dependent metabolic defects. Importantly, lowering T cell–intrinsic ROS restricted SENP7 cytosolic translocation and repressed CD8^+^ T cell metabolic and functional activity in human colorectal cancer samples. Our findings reveal that SENP7, as an oxidative stress sensor, sustains CD8^+^ T cell metabolic fitness and effector functions and unveil an oxidative stress–sensing machinery in tumor-infiltrating CD8^+^ T cells.

## Graphical abstract

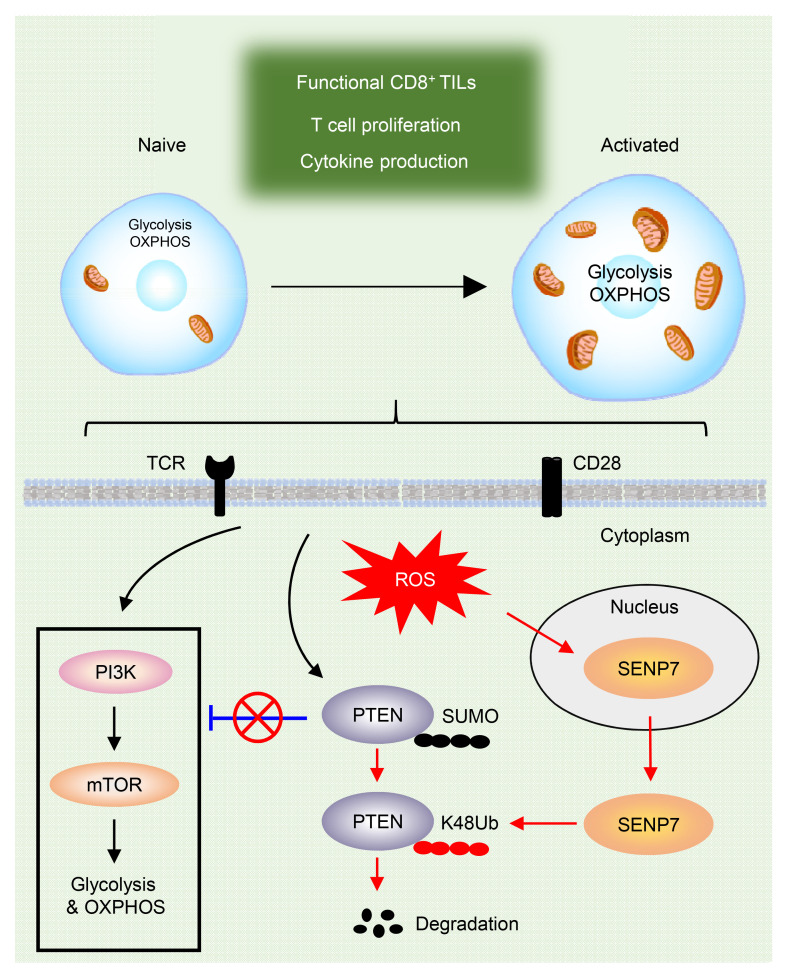

## Introduction

CD8^+^ T cells mediate potent antitumor functions and play a critical role in immunotherapy-induced immune responses against cancer (1–3). Functionality and metabolic fitness are central determinants of CD8^+^ T cell efficacy in cancer immunotherapy (4). Importantly, the functional integrity of CD8^+^ T cells is tightly coupled to metabolic reprogramming (5–7). Proliferating CD8^+^ T cells not only utilize aerobic glycolysis to support the rapid production of ATP but also engage mitochondrial metabolism to generate sufficient amounts of bioenergetic intermediates and fulfill bioenergetic and biosynthetic demands (4, 8, 9). However, tumor-infiltrating CD8^+^ T cells typically undergo metabolic exhaustion, which is associated with T cell dysfunction, in the tumor microenvironment (TME) (10–13). The mechanism by which CD8^+^ T cell metabolic and functional activity is metabolically regulated in the TME remains unclear.

Oxidative stress, which is reflected by elevated levels of ROS in the TME, is a hallmark of many cancers (14, 15). Physiological levels of ROS are important for T cell activation, expansion, and effector function (16–19). Specific ROS-dependent signaling events are needed for T cell activation in vitro and antigen-specific T cell expansion in vivo (18). Persistent dysfunction of oxidative metabolism leads to impaired T cell antitumor functions in vivo (20). On the other hand, increased ROS production induced by mitochondrial dysfunction drives T cell exhaustion, and ROS neutralization slows T cell terminal differentiation (21). Notably, the mechanism by which oxidative stress shapes CD8^+^ T cell metabolic and functional fitness in the TME remains elusive. Clarifying the oxidative stress–sensing machinery of tumor-infiltrating CD8^+^ T cells would be valuable.

SUMOylation is a reversible posttranslational modification (PTM) and regulates the subcellular localization, stability, and transcriptional activity of target proteins (22, 23). Notably, SUMOylation acts as a sensitive sensor of redox species and is strongly connected with oxidative stress (24). We and others have shown that SUMO-specific protease 3 (SENP3) is a redox sensor involved in multiple physiological and pathological processes (15, 25–27). Cytosolic accumulation of SENP3 is induced to enhance autophagosome formation in a ROS-dependent manner upon cellular starvation (28). Moreover, ROS-induced SENP3 protein stability is responsible for regulatory T cell maintenance and DC function in the TME (15, 27). SUMO-specific protease 7 (SENP7), another SUMO2/3-specific protease, is known for regulating epithelial-mesenchymal transition, DNA repair, and innate immune responses (29–32). However, whether SENP7 senses oxidative stress to affect the phenotype and function of tumor-infiltrating CD8^+^ T cells has not previously been defined.

Here, we present biochemical, genetic, and functional evidence that SENP7, as an oxidative stress sensor, sustains CD8^+^ T cell metabolic and functional states. We found that ROS-induced cytosolic translocation of SENP7 promoted PTEN degradation and prevented PTEN-dependent metabolic defects, thereby enhancing CD8^+^ T cell antitumor function. Accordingly, pharmacological targeting of ROS dampened the metabolic fitness of intratumoral CD8^+^ T cells and impaired their effector functions. These findings highlight the oxidative stress–sensing machinery of CD8^+^ T cells in the TME and suggest that targeting this process may improve cancer immunotherapy.

## Results

### ROS trigger cytosolic translocation of SENP7 in tumor-infiltrating CD8^+^ T cells.

**Figure 1:**
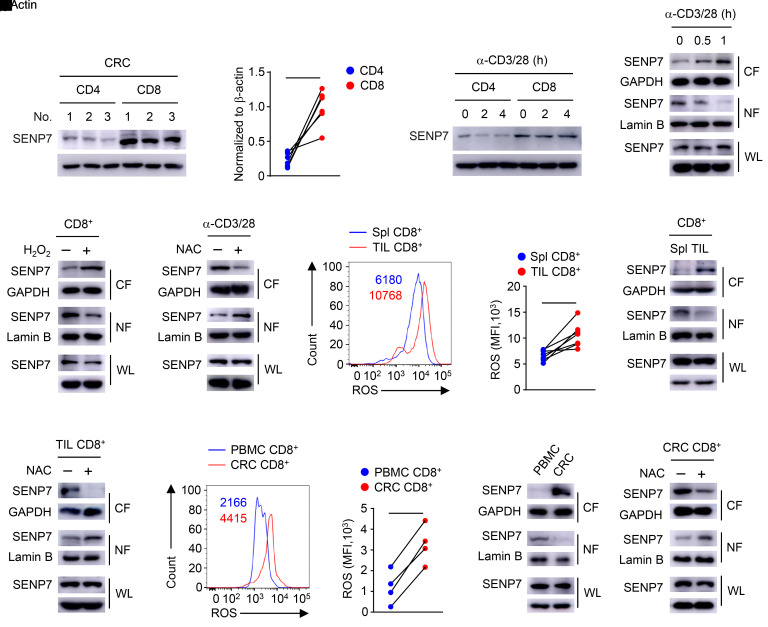
ROS trigger cytosolic translocation of SENP7 in tumor-infiltrating CD8^+^ T cells. (**A** and **B**) Immunoblot analysis of the indicated proteins (**A**) and quantification of SENP7 expression (**B**, *n =* 6) in CD4^+^ and CD8^+^ T cells from CRC tissues. (**C**) Immunoblot analysis of the indicated proteins in CD4^+^ and CD8^+^ T cells from WT mice stimulated with anti-CD3 and anti-CD28 antibodies (α-CD3/28). (**D**–**F**) Immunoblot analysis using whole-cell lysates (WL) and nuclear (NF) and cytoplasmic (CF) fractions of CD8^+^ T cells from WT mice stimulated with anti-CD3 and anti-CD28 antibodies (**D**), CD8^+^ T cells from WT mice treated with 0.2 mM H_2_O_2_ for 1 hour (**E**), and CD8^+^ T cells from WT mice stimulated with anti-CD3 and anti-CD28 antibodies plus 10 mM NAC for 1 hour (**F**). (**G** and **H**) Histogram shows the MFI of ROS (**G**) and quantification of the MFI of ROS (**H**, *n =* 7) in CD8^+^ T cells from the spleens (Spl) and tumors (TIL) of tumor-bearing mice (day 7 after injection of tumors with MC38 cells). (**I** and **J**) Immunoblot analysis using CD8^+^ T cells from the spleens and tumors of tumor-bearing mice (**I**) and tumor-infiltrating CD8^+^ T cells treated with 10 mM NAC for 1 hour (**J**). (**K** and **L**) Histogram shows the MFI of ROS (**K**) and quantification of the MFI of ROS (**L**, *n =* 4) in CD8^+^ T cells from patient-derived PBMCs and CRC tissues. (**M** and **N**) Immunoblot analysis of the indicated proteins in CD8^+^ T cells from patient-derived PBMCs and CRC tissues (**M**) and CD8^+^ T cells from CRC tissues treated with 10 mM NAC for 1 hour (**N**). Representative data are shown from 2 (**A**, **M**, and **N**) and 3 (**C**–**F**, **I**, and **J**) independent experiments. *P < 0.05 and **P < 0.01, by Student’s *t* test (**B**, **H** and **L**).

To investigate the expression profile of SENP7 in tumor-infiltrating T cells, we isolated CD4^+^ T cells and CD8^+^ T cells from patient-derived colorectal cancer (CRC) tissue for immunoblot analysis. Intriguingly, SENP7 was highly accumulated in tumor-infiltrating CD8^+^ T cells (Figure 1, A and B). Similarly, SENP7 expression was greatly increased in CD8^+^ T cells from WT mice (Figure 1C). Notably, SENP7 cytosolic translocation was induced in CD8^+^ T cells upon activation by the TCR signals, although SENP7 protein levels were not altered (Figure 1D). Since TCR signals trigger ROS production in CD8^+^ T cells (33), we sought to determine the potential connection of ROS with SENP7 cytosolic translocation. Addition of H_2_O_2_ promoted the cytosolic translocation of SENP7 in CD8^+^ T cells (Figure 1E). Incubation of activated CD8^+^ T cells with *N*-acetylcysteine (NAC), a well-defined ROS inhibitor, prevented TCR-induced cytosolic translocation of SENP7 (Figure 1F). Furthermore, tumor-infiltrating CD8^+^ T cells exhibited higher levels of ROS and cytosolic SENP7 than did splenic CD8^+^ T cells from tumor-bearing mice (Figure 1, G–I). In response to NAC treatment, SENP7 in tumor-infiltrating CD8^+^ T cells did not localize to the cytoplasm (Figure 1J). Importantly, the levels of ROS and cytosolic SENP7 in CD8^+^ T cells from human CRC tissue were higher than those in CD8^+^ T cells from PBMCs (Figure 1, K–M). Pharmacologic inhibition of ROS prevented SENP7 cytosolic translocation in CD8^+^ T cells from CRC tissue (Figure 1N). Collectively, these results indicate that ROS trigger SENP7 cytosolic translocation in tumor-infiltrating CD8^+^ T cells.

### SENP7 ablation dampens CD8^+^ T cell antitumor responses in vivo.

**Figure 2:**
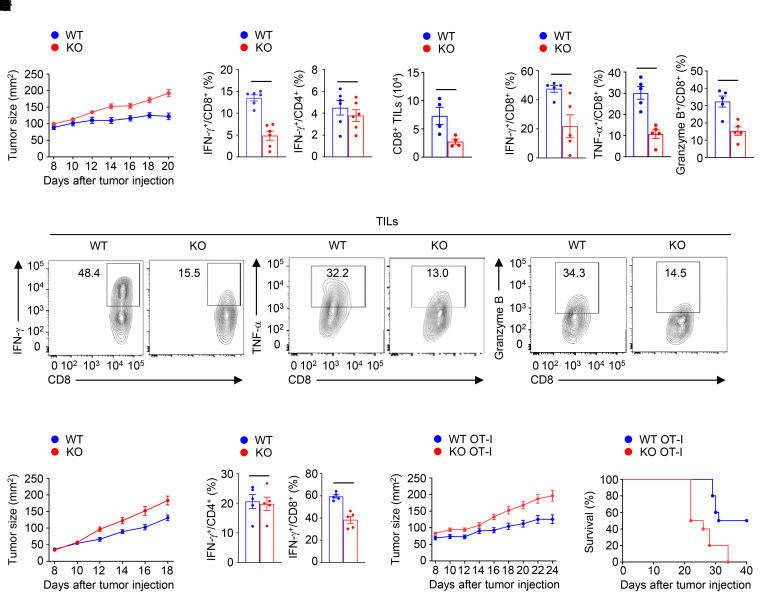
SENP7 ablation dampens CD8^+^ T cell antitumor responses in vivo. (**A**) Tumor growth in *Senp7^fl/fl^* (WT) and *Senp7^fl/fl^* *Cd4*-Cre (KO) mice injected s.c. with MC38 murine colon cancer cells (*n =* 10 mice per group). (**B**) Flow cytometric analysis of the frequency of IFN-γ–producing CD8^+^ or CD4^+^ T cells in the draining lymph nodes of WT and KO mice injected s.c. with MC38 murine colon cancer cells (day 14, *n =* 6). (**C**) CD8^+^ T cell numbers in tumors (TILs) of WT and KO mice injected s.c. with MC38 murine colon cancer cells (day 14, *n =* 4) were normalized to 100 mg tumor tissue. (**D** and **E**) Flow cytometric analysis of IFN-γ–producing, TNF-α–producing, or granzyme B–producing CD8^+^ T cells in the tumors of WT and KO mice injected s.c. with MC38 murine colon cancer cells (day 14, *n =* 5). The data are presented as summary graphs in **D** and as representative plots in **E**. (**F**) Tumor growth in WT and KO mice injected s.c. with B16-F10 melanoma cells (*n =* 10 mice per group). (**G**) Frequency of IFN-γ–producing CD4^+^ T cells or CD8^+^ T cells in the tumors of WT and KO mice injected s.c. with B16-F10 melanoma cells (day 14, *n =* 5). (**H** and **I**) Tumor growth and survival curves for B6.SJL mice injected s.c. with MC38-OVA cancer cells adoptively transferred with WT OT-I or *Senp7^fl/fl^* *Cd4*-Cre (KO) OT-I CD8^+^ T cells on day 7 after tumor cell inoculation (*n =* 10 mice per group). Representative data are shown from 3 independent experiments. Data are presented as the mean ± SEM. *P < 0.05 and **P < 0.01, by 2-tailed Student’s *t* test (**A**–**D** and **F**–**H**) and log-rank (Mantel-Cox) test (**I**).

To explore the role of SENP7 in CD8^+^ T cell antitumor function, we crossed *Senp7^fl/fl^* mice with *Cd4*-Cre mice to obtain *Senp7^fl/fl^* (designated WT) and *Senp7^fl/fl^* *Cd4*-Cre (designated KO) mice (Supplemental Figure 1A; supplemental material available online with this article; https://doi.org/10.1172/JCI155224DS1). The KO mice did not show obvious abnormalities in thymocyte development or peripheral T cell frequency (Supplemental Figure 1, B–D). In addition, the percentages of Tregs in the thymus, spleen, and lymph nodes were comparable between WT and KO mice (Supplemental Figure 1E). Since CD8^+^ T cells play a crucial role in cell-mediated tumor killing (34), we inoculated WT and KO mice with MC38 murine colon cancer cells to determine whether SENP7 is required for antitumor T cell immune responses in vivo. Compared with WT mice, KO mice exhibited a profound increase in tumor size (Figure 2A). CD8^+^ T cells in the draining lymph nodes from tumor-bearing KO mice produced less IFN-γ than did those from tumor-bearing WT mice, whereas the frequencies of IFN-γ–producing CD4^+^ T cells in the draining lymph nodes were similar between tumor-bearing KO mice and tumor-bearing WT mice (Figure 2B). The tumor-bearing KO mice displayed decreased numbers of tumor-infiltrating CD8^+^ T cells (Figure 2C). Moreover, the tumor-bearing KO mice had decreased frequencies of tumor-infiltrating IFN-γ–producing, TNF-α–producing, and granzyme B–producing CD8^+^ T cells (Figure 2, D and E). By contrast, the frequencies of IFN-γ–producing CD4^+^ T cells in the tumors were similar between tumor-bearing KO mice and tumor-bearing WT mice (Supplemental Figure 2A). Parallel studies revealed that the KO mice also displayed impaired CD8^+^ T cell–mediated antitumor immunity in the B16-F10 murine melanoma model (Figure 2, F and G). To further confirm the CD8^+^ T cell–specific role of SENP7 in antitumor function, we adoptively transferred OT-I cells isolated from *Senp7^fl/fl^* *Cd4*-Cre OT-I (KO OT-I) mice and their WT OT-I littermates into WT mice bearing chicken OVA–expressing MC38 (MC38-OVA) tumors. As expected, mice transferred with KO OT-I T cells displayed a weaker suppression of tumor growth and tumor-induced lethality (Figure 2, H and I). However, the tumor size and tumor-induced lethality were similar between the recipients of WT OT-II cells and KO OT-II cells (Supplemental Figure 2, B and C), indicating that SENP7 is dispensable for CD4^+^ T cell antitumor function. These results suggest a CD8^+^ T cell–specific role of SENP7 in antitumor function in vivo.

### SENP7 is indispensable for CD8^+^ T cell proliferation in vivo and in vitro.

**Figure 3:**
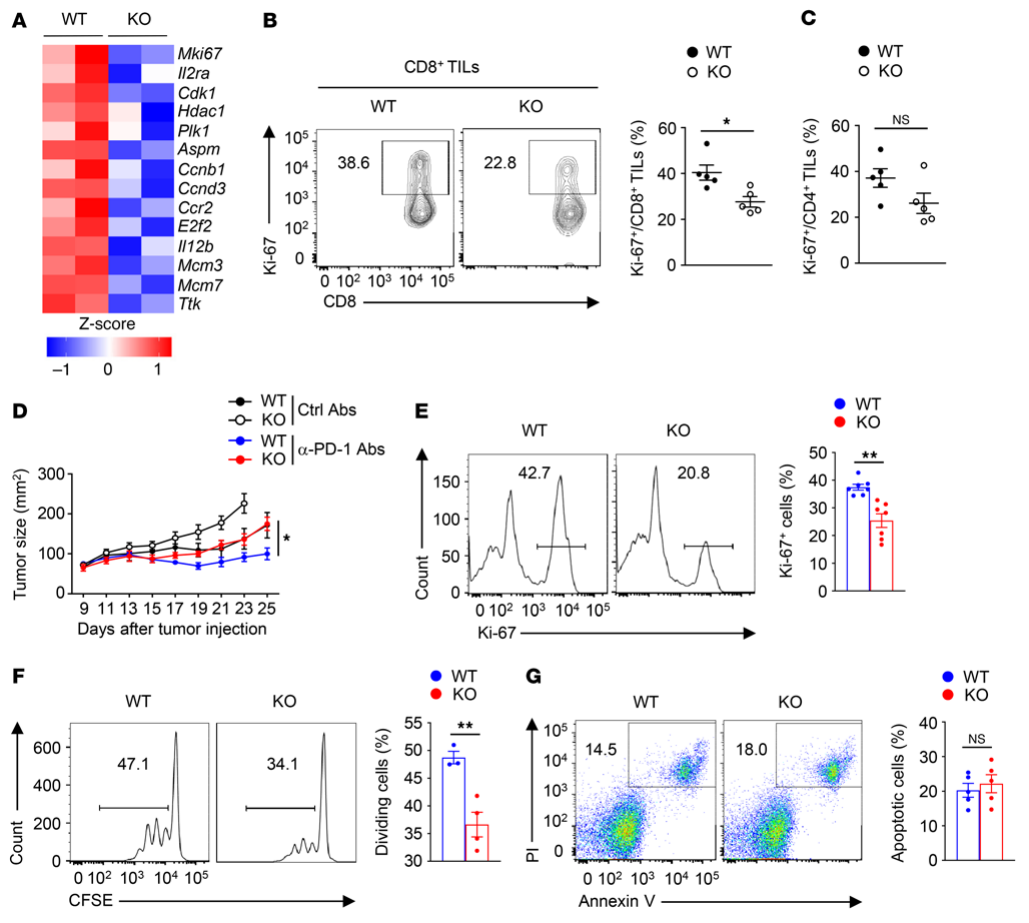
SENP7 is indispensable for CD8^+^ T cell proliferation in vivo and in vitro. (**A**) Heatmap of downregulated genes associated with T cell proliferation in tumor-infiltrating WT and SENP7-deficient CD8^+^ T cells. CD8^+^ T cells were isolated from tumor-bearing WT and KO mice on day 7 after injection of tumors with MC38 murine colon cancer cells. (**B** and **C**) Flow cytometric analysis of the frequency of Ki-67^+^ tumor-infiltrating CD8^+^ T cells (**B**) and CD4^+^ T cells (**C**) from WT and KO mice injected s.c. with MC38 murine colon cancer cells (day 14, *n =* 5). (**D**) Tumor growth in WT and KO mice injected with MC38 colon cancer cells (*n =* 6) followed by i.p. injection with 50 μg anti–PD-1 antibody or control antibody (Ctrl) on days 7, 10, and 13. (**E**) Flow cytometric analysis of the frequency of Ki-67^+^ WT and KO CD8^+^ T cells stimulated with anti-CD3 and anti-CD28 antibodies for 2 days (*n =* 7). (**F**) Flow cytometric analysis of the division of WT and KO CD8^+^ T cells. Naive WT and KO CD8^+^ T cells labeled with CFSE were stimulated for 72 hours with antibodies against CD3 and CD28. (**G**) Flow cytometric analysis of apoptotic WT and KO CD8^+^ T cells stimulated with anti-CD3 and anti-CD28 antibodies for 1 day (*n =* 5). Data are representative of 3 or more independent experiments and are presented as the mean ± SEM. *P < 0.05 and **P < 0.01, by 2-way ANOVA with Geisser-Greenhouse correction (**D**) and 2-tailed Student’s *t* test (**B**, **C**, and **E**–**G**).

To clarify the mechanism underlying the reduced antitumor activity of SENP7-deficient CD8^+^ T cells, we isolated tumor-infiltrating CD8^+^ T cells from tumor-bearing WT and KO mice for transcriptomic analysis. Cell proliferation–related genes, including *Mki67*, *Cdk1*, *Plk1*, and *Ccnb1*, were downregulated in tumor-infiltrating SENP7-deficient CD8^+^ T cells (Figure 3A). Consistent with this finding, the percentage of Ki-67^+^ tumor-infiltrating CD8^+^ T cells was lower in tumor-bearing KO mice (Figure 3B). By contrast, tumor-bearing WT and KO mice had similar percentages of Ki-67^+^ tumor-infiltrating CD4^+^ T cells (Figure 3C). Although anti–programmed cell death 1 (anti–PD-1) treatment efficiently inhibited tumor progression in tumor-bearing WT mice, this efficiency was largely suppressed in tumor-bearing KO mice (Figure 3D), indicating that SENP7-dependent CD8^+^ T cell proliferation contributed to the antitumor immune response to checkpoint blockade.

To confirm that SENP7 is required for CD8^+^ T cell proliferation, we examined CD8^+^ T cell activity in vitro. Indeed, expression of the cell proliferation marker Ki-67 after TCR and CD28 stimulation was attenuated in SENP7-deficient CD8^+^ T cells (Figure 3E). Consistent with this finding, defective proliferation of SENP7-deficient CD8^+^ T cells was observed after TCR and CD28 stimulation (Figure 3F). On the other hand, neither the induction of T cell activation markers (Supplemental Figure 3, A and B) nor the frequency of apoptotic cells (Figure 3G) was affected in SENP7-deficient CD8^+^ T cells. Furthermore, the expression of Ki-67 in SENP7-deficient CD4^+^ T cells was similar to that in WT CD4^+^ T cells (Supplemental Figure 3C). Therefore, SENP7 is indispensable for CD8^+^ T cell proliferation in vivo and in vitro.

### SENP7 promotes glycolysis and oxidative phosphorylation in CD8^+^ T cells.

**Figure 4:**
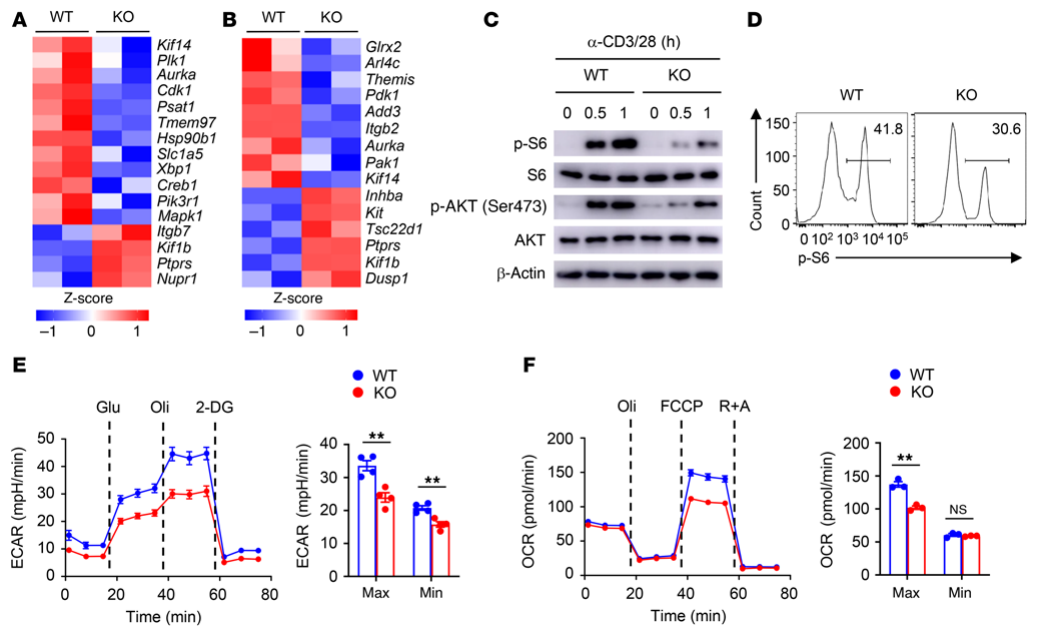
SENP7 promotes glycolysis and OXPHOS of CD8^+^ T cells. (**A**) RNA-Seq analysis using WT and KO CD8^+^ T cells isolated from the tumors of mice injected s.c. with MC38 colon cancer cells (day 7 after injection). Heatmap shows PI3K/mTOR signaling pathway–related genes. (**B**) RNA-Seq analysis using naive WT and KO CD8^+^ T cells stimulated with anti-CD3 and anti-CD28 antibodies for 8 hours. Heatmap shows PI3K/mTOR signaling pathway–related genes. (**C**) Immunoblot analysis of the indicated proteins in WT and KO CD8^+^ T cells stimulated with anti-CD3 and anti-CD28 antibodies for the indicated durations. (**D**) Flow cytometric analysis of p-S6 expression in WT and KO CD8^+^ T cells stimulated with anti-CD3 and anti-CD28 antibodies for 2 hours. (**E**) ECAR of WT and KO naive CD8^+^ T cells stimulated for 6 hours with antibodies against CD3 and CD28 under minimum (Min) or maximum (Max) conditions with the addition of different reagents (*n =* 4). (**F**) OCR of WT and KO naive CD8^+^ T cells stimulated for 6 hours with antibodies against CD3 and CD28 under minimum or maximum conditions with the addition of different reagents (*n =* 3). Data are representative of 3 independent experiments and are presented as the mean ± SEM. **P < 0.01, by Student’s *t* test. Glu, glucose; Oli, oligomycin; R+A, rotenone/antimycin A.

To explore SENP7-dependent transcriptional programs, we analyzed the gene expression profiles of tumor-infiltrating CD8^+^ T cells. Intriguingly, the phosphoinositide 3-kinase/mTOR (PI3K/mTOR) signaling pathway–related gene expression profile was altered in tumor-infiltrating SENP7-deficient CD8^+^ T cells (Figure 4A). We further performed RNA-Seq using naive CD8^+^ T cells activated in vitro to confirm the gene expression profiles. As expected, the levels of genes involved in the PI3K/mTOR signaling pathway were modulated in SENP7-deficient CD8^+^ T cells (Figure 4B). Immunoblot and flow cytometric analyses revealed that activation of mTORC1 (assessed by measuring the phosphorylation of ribosomal protein S6) and mTORC2 (assessed by measuring the phosphorylation of AKT at Ser473) was impaired in SENP7-deficient CD8^+^ T cells stimulated with anti-CD3 and anti-CD28 antibodies (Figure 4, C and D). Because the PI3K/mTOR signaling pathway mediates metabolic reprogramming to sustain T cell proliferation and effector function (34), we next assessed CD8^+^ T cell metabolic fitness. Interestingly, SENP7-deficient CD8^+^ T cells had significantly lower baseline and maximum glycolytic rates than did WT CD8^+^ T cells (Figure 4E). Moreover, SENP7-deficient CD8^+^ T cells displayed decreased oxidative phosphorylation (OXPHOS) rates at maximum capacity (Figure 4F). Hence, SENP7 promotes activation of PI3K/mTOR signaling to sustain CD8^+^ T cell glycolysis and OXPHOS.

### SENP7-dependent reduction in PTEN controls CD8^+^ T cell metabolism and function.

**Figure 5:**
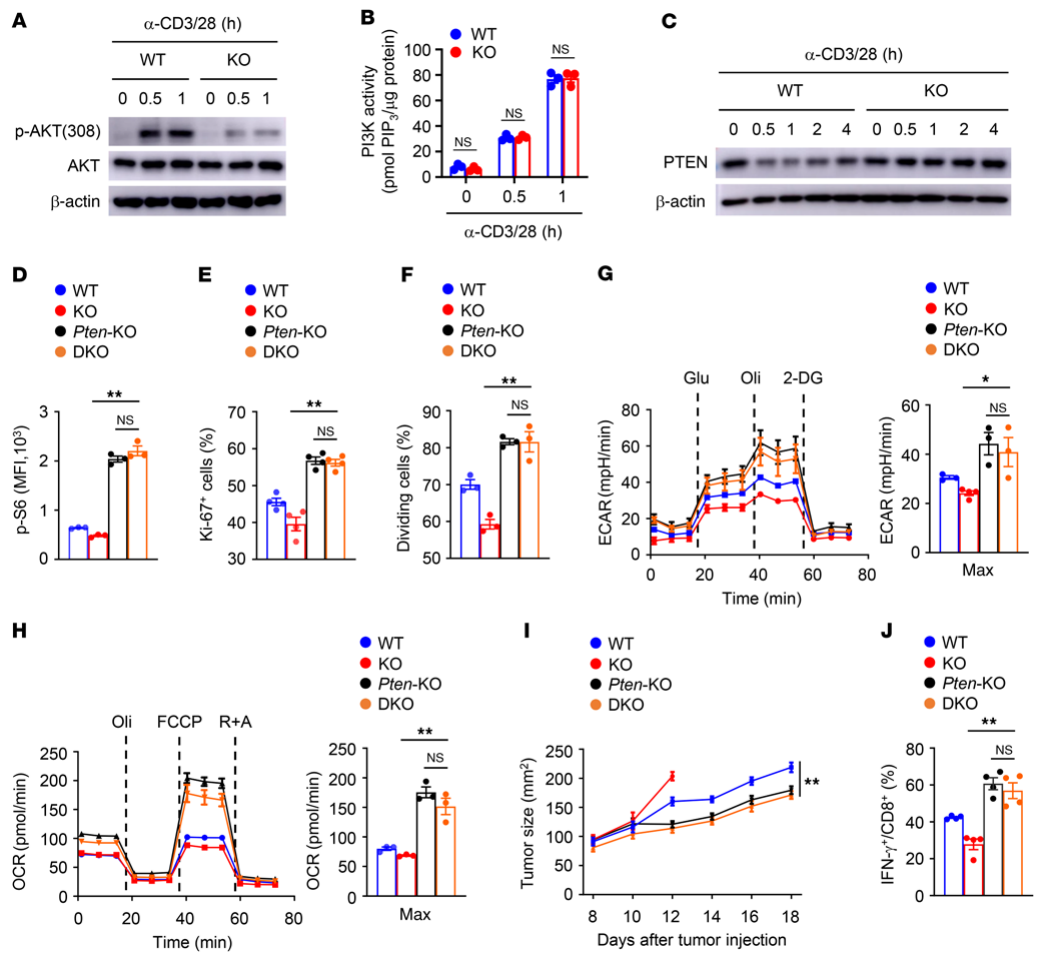
SENP7-dependent reduction of PTEN controls CD8^+^ T cell metabolism and function. (**A**) Immunoblot analysis of the indicated proteins in WT and KO CD8^+^ T cells after stimulation with anti-CD3 and anti-CD28 antibodies. (**B**) PI3K activity in WT and KO CD8^+^ T cells stimulated with anti-CD3 and anti-CD28 antibodies was analyzed using an Echelon kit (*n =* 3). PIP_3_, phosphatidylinositol (3,4,^5^)-trisphosphate. (**C**) Immunoblot analysis of the indicated proteins in WT and KO CD8^+^ T cells after stimulation with anti-CD3 and anti-CD28 antibodies. (**D** and **E**) Flow cytometric analysis of p-S6 (**D**) and Ki-67 (**E**) expression in WT, *Senp7^fl/fl^* *Cd4*-Cre (KO), *Pten^fl/fl^* *Cd4*-Cre (*Pten*-KO), and *Pten^fl/fl^* *Senp7^fl/fl^* *Cd4*-Cre (DKO) CD8^+^ T cells stimulated with anti-CD3 and anti-CD28 antibodies for 2 hours (**D**, *n =* 3) or 2 days (**E**, *n =* 4). (**F**) Flow cytometric analysis of the division of WT, KO, *Pten*-KO, and DKO CD8^+^ T cells labeled with CFSE upon stimulation with anti-CD3 and anti-CD28 antibodies for 72 hours (*n =* 3). (**G** and **H**) ECAR (**G**) and OCR (**H**) of WT, KO, *Pten*-KO, and DKO CD8^+^ T cells stimulated for 6 hours with anti-CD3 and anti-CD28 antibodies. (**I**) Tumor growth of MC38 tumor–bearing *Rag1*-KO mice injected with 2 × 10^6^ WT, KO, *Pten*-KO, or DKO CD8^+^ T cells along with 2 × 10^6^ WT CD4^+^ T cells on day 7 after tumor cell inoculation (*n =* 10). (**J**) Flow cytometric analysis of IFN-γ–producing CD8^+^ T cells in the tumors of *Rag1*-KO mice from **I** (day 12, *n =* 4). Data shown are representative of 3 independent experiments and are presented as the mean ± SEM. *P < 0.05 and **P < 0.01, by 1-way ANOVA with Tukey’s multiple-comparison test (**D**–**H** and **J**); 2-way ANOVA with Geisser-Greenhouse correction (**I**); and 2-tailed Student’s *t* test (**B**).

Our results revealed that SENP7 deficiency inhibited phosphorylation of AKT at Thr308 in CD8^+^ T cells (Figure 5A), indicating that the upstream pathway of PI3K/mTOR signaling is regulated by SENP7. However, PI3K activity was comparable in WT and KO CD8^+^ T cells (Figure 5B). Since PTEN serves as the main negative regulator of PI3K (35), we next examined the activity of PTEN. Interestingly, TCR and CD28 stimulation led to a reduction of PTEN protein expression in WT CD8^+^ T cells (Figure 5C). Notably, SENP7 deficiency strongly prevented the T cell receptor– (TCR-) and CD28-induced reduction in PTEN expression (Figure 5C). To validate the contribution of PTEN activity to the observed phenotypes of SENP7-deficient CD8^+^ T cells, we crossed *Senp7^fl/fl^* *Cd4*-Cre (KO) mice with *Pten^fl/fl^* mice to generate *Senp7^fl/fl^* *Pten^fl/fl^* *Cd4*-Cre (double-KO [DKO]) mice. Compared with stimulated *Senp7^fl/fl^* *Cd4*-Cre (KO) CD8^+^ T cells, stimulated DKO CD8^+^ T cells showed markedly enhanced mTORC1 activity (Figure 5D). Ki-67 expression and the proliferation of activated SENP7-deficient CD8^+^ T cells were considerably augmented by deletion of PTEN (Figure 5, E and F). Furthermore, the glycolytic and OXPHOS rates in activated SENP7-deficient CD8^+^ T cells were considerably increased by deletion of PTEN (Figure 5, G and H). Importantly, SENP7-sufficient and SENP7-deficient CD8^+^ T cells displayed comparable levels of S6 phosphorylation, Ki-67 expression, cell proliferation, glycolysis, and OXPHOS in the absence of PTEN (Figure 5, D–H). We further reconstituted *Rag1*-KO mice with WT, *Senp7^fl/fl^* *Cd4*-Cre (KO), *Pten^fl/fl^* *Cd4*-Cre (*Pten*-KO), or *Pten^fl/fl^* *Senp7^fl/fl^* *Cd4*-Cre (DKO) CD8^+^ T cells along with an equal number of WT CD4^+^ T cells. After MC38 tumor challenge, *Rag1*-KO mice reconstituted with PTEN-deficient CD8^+^ T cells plus WT CD4^+^ T cells had enhanced antitumor immunity (Figure 5, I and J). Importantly, the tumor size and frequency of IFN-γ–producing CD8^+^ T cells in tumors were similar between the recipients of DKO CD8^+^ T cells plus WT CD4^+^ T cells and the recipients of *Pten*-KO CD8^+^ T cells plus WT CD4^+^ T cells (Figure 5, I and J). These results suggest that a SENP7-dependent reduction in PTEN maintains CD8^+^ T cell metabolic fitness and effector function.

### SENP7-mediated PTEN deSUMOylation facilitates PTEN degradation.

**Figure 6:**
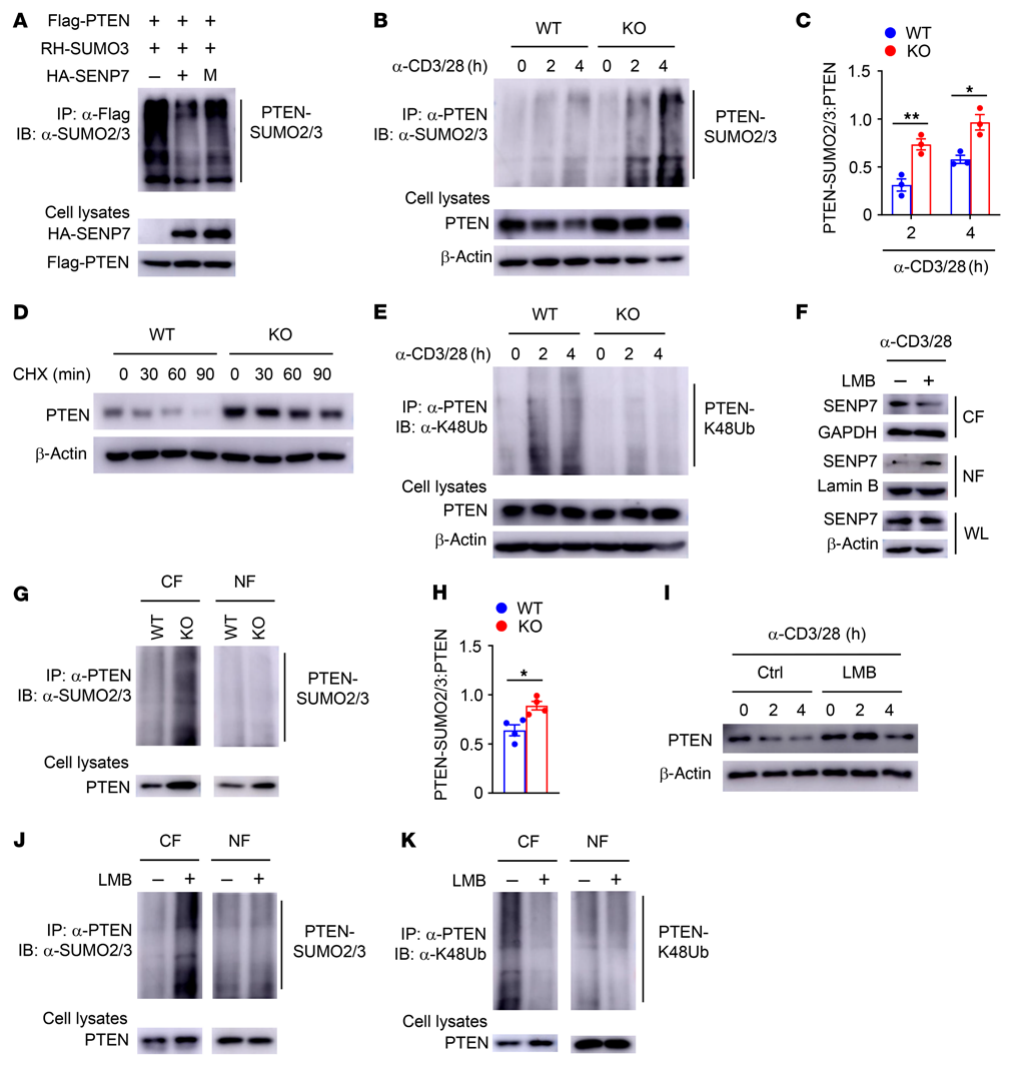
SENP7-mediated PTEN deSUMOylation facilitates PTEN degradation. (**A**) HEK293T cells cotransfected with Flag-tagged PTEN, Ubc9, and RH-SUMO3 in the presence of WT or catalytically inactive (C979S) mutant (M) SENP7 were immunoprecipitated with anti-Flag antibody and assessed by immunoblotting (IB) with anti-SUMO2/3. HA, hemagglutinin; RH, arginine(R)-glycine(G)-serine(S)-histidine. (**B**) PTEN SUMOylation assays were performed by immunoprecipitating PTEN under denaturing conditions followed by detection of SUMOylated PTEN using anti-SUMO2/3 antibodies in CD8^+^ T cells stimulated with anti-CD3 and anti-CD28 antibodies. (**C**) Quantifications of PTEN-SUMO2/3:PTEN levels in CD8^+^ T cells stimulated with anti-CD3 and anti-CD28 antibodies (*n =* 3). (**D**) Immunoblot analysis of the indicated proteins in CD8^+^ T cells stimulated with anti-CD3 and anti-CD28 antibodies for 1 hour following incubation with CHX (50 μg/mL) for the indicated durations. (**E**) PTEN ubiquitination assays in CD8^+^ T cells stimulated with anti-CD3 and anti-CD28 antibodies in the presence of MG132. (**F**) Immunoblot analysis of the indicated proteins in CD8^+^ T cells from WT mice stimulated with anti-CD3 and anti-CD28 antibodies plus LMB for 2 hours. (**G**) PTEN SUMOylation assays using nuclear and cytoplasmic fractions of WT and KO CD8^+^ T cells stimulated with anti-CD3 and anti-CD28 antibodies for 2 hours. (**H**) Quantification of PTEN-SUMO2/3:PTEN levels in the cytoplasmic fractions of WT and KO CD8^+^ T cells stimulated with anti-CD3 and anti-CD28 antibodies for 2 hours (*n =* 4). (**I**) Immunoblot analysis of the indicated proteins in CD8^+^ T cells from WT mice stimulated with anti-CD3 and anti-CD28 antibodies plus LMB for 0, 2, and 4 hours. (**J** and **K**) PTEN SUMOylation (**J**) and ubiquitination (**K**) assays using nuclear and cytoplasmic fractions of CD8^+^ T cells from WT mice stimulated with anti-CD3 and anti-CD28 antibodies plus LMB for 2 hours. Data are representative of 3 independent experiments and presented as the mean ± SEM. *P < 0.05 and **P < 0.01, by Student’s *t* test.

We next sought to determine whether SENP7 mediates deSUMOylation to regulate PTEN activity. SENP7 was found to interact with PTEN in CD8^+^ T cells (Supplemental Figure 4A). Transfection of human embryonic kidney 293T (HEK293T) cells with the corresponding constructs further confirmed the interaction between SENP7 and PTEN (Supplemental Figure 4B). When we coexpressed PTEN with SENP7, PTEN SUMOylation was dramatically inhibited, but this effect did not occur with coexpression of the catalytically inactive SENP7 (Figure 6A). Consistent with this finding, PTEN was transiently SUMOylated in KO CD8^+^ T cells upon TCR and CD28 stimulation, but this effect was not observed in WT CD8^+^ T cells (Figure 6, B and C), indicating that SENP7 prevents the SUMOylation of PTEN in activated CD8^+^ T cells. Treatment with a protein synthesis inhibitor, cycloheximide (CHX), led to substantial loss of PTEN in activated WT CD8^+^ T cells but not in activated KO CD8^+^ T cells (Figure 6D), indicating that SENP7 regulates PTEN degradation. Because K48-linked ubiquitin chains are the primary signal for protein degradation (36), we next examined the level of K48-linked ubiquitination (K48Ub) of PTEN in activated CD8^+^ T cells. Blocking protein degradation with a proteasome inhibitor, MG132, resulted in accumulation of PTEN in WT CD8^+^ T cells, similar to that observed in SENP7-deficient CD8^+^ T cells (Figure 6E). In this condition, we observed abundant K48-linked ubiquitination of PTEN in activated WT CD8^+^ T cells (Figure 6E), and this modification is likely responsible for TCR- and CD28-induced PTEN degradation. However, this phenomenon did not occur in activated SENP7-deficient CD8^+^ T cells (Figure 6E), indicating that dramatically increased PTEN SUMOylation inhibits the K48-linked ubiquitination and degradation of PTEN in activated SENP7-deficient CD8^+^ T cells. On the other hand, loss of SENP7 did not change the SUMOylation status or stability of PTEN in activated CD4^+^ T cells (Supplemental Figure 4C), which is most likely due to the low levels of SENP7 (Figure 1C).

We further examined the potential connection of TCR-triggered PTEN degradation with TCR-induced SENP7 cytosolic translocation. In response to treatment with leptomycin B (LMB), an inhibitor of nuclear export, TCR signal–induced SENP7 cytosolic translocation was largely blocked in WT CD8^+^ T cells (Figure 6F). We observed abundant SUMOylation of PTEN in the cytoplasm but not the nucleus of activated SENP7-deficient CD8^+^ T cells (Figure 6, G and H). Notably, PTEN stability was greatly restored in activated WT CD8^+^ T cells after the treatment with LMB (Figure 6I). Moreover, LMB treatment markedly increased the SUMOylation and reduced the K48-linked ubiquitination of PTEN in the cytoplasm but not the nucleus of activated WT CD8^+^ T cells (Figure 6, J and K). By contrast, LMB treatment did not alter the stability, SUMOylation, or K48-linked ubiquitination of PTEN in activated SENP7-deficient CD8^+^ T cells (Supplemental Figure 4, D–F). These data indicate that cytosolic SENP7 mediates PTEN deSUMOylation to facilitate the K48-linked ubiquitination and degradation of PTEN in activated CD8^+^ T cells.

### ROS are required for SENP7-dependent CD8^+^ T cell metabolism and function.

**Figure 7:**
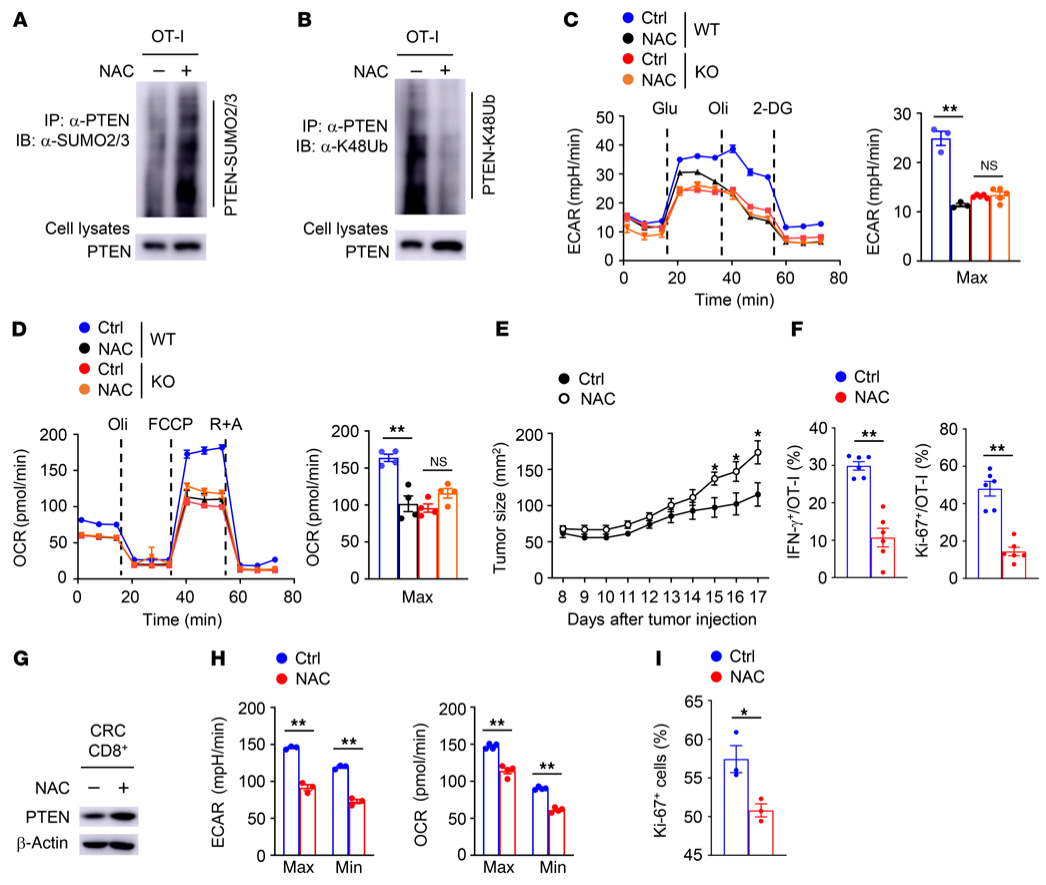
ROS involve SENP7-dependent CD8^+^ T cell metabolism and function. (**A** and **B**) PTEN SUMOylation (**A**) and ubiquitination (**B**) assays using WT OT-I cells stimulated with anti-CD3 and anti-CD28 antibodies plus NAC for 2 hours. (**C** and **D**) ECAR (**C**) and OCR (**D**) of WT and KO OT-I cells stimulated with anti-CD3 and anti-CD28 antibodies plus NAC for 6 hours. (**E**) Tumor growth of MC38-OVA tumor–bearing WT mice (day 6 after tumor cell inoculation) injected i.v. with WT OT-I cells stimulated with anti-CD3 and anti-CD28 antibodies plus 10 mM NAC for 8 hours in vitro (control: *n =* 8; NAC: *n =* 9). (**F**) Flow cytometric analysis of the frequency of IFN-γ–producing and Ki-67^+^ OT-I cells in the tumors of mice from **E** (day 17 after tumor injection, *n =* 6). (**G**) Immunoblot analysis of the indicated proteins in CD8^+^ T cells from CRC samples incubated in vitro for 2 hours in complete media containing 10 mM NAC. (**H**) ECAR and OCR of CD8^+^ T cells from CRC samples incubated in vitro for 2 hours in complete media containing 10 mM NAC. (**I**) Flow cytometric analysis of the frequency of Ki-67^+^ CD8^+^ T cells from CRC samples incubated in vitro for 6 hours in complete media containing 10 mM NAC (*n =* 3). Data are representative of 3 independent experiments and presented as the mean ± SEM. *P < 0.05 and **P < 0.01, by 1-way ANOVA with Tukey’s multiple-comparison test (**C** and **D**) and 2-tailed Student’s *t* test (**E**, **F**, **H**, and **I**).

To assess whether ROS are involved in the regulation of SENP7-dependent CD8^+^ T cell metabolism and function, we treated WT and SENP7-deficient OT-I cells with NAC. NAC treatment substantially increased the SUMOylation and reduced the K48-linked ubiquitination of PTEN in WT OT-I cells (Figure 7, A and B). Furthermore, NAC-treated WT OT-I cells exhibited decreased glycolysis and OXPHOS (Figure 7, C and D). To explore the role of ROS in CD8^+^ T cell antitumor function, WT OT-I cells stimulated with anti-CD3 and anti-CD28 antibodies plus NAC in vitro were transferred into tumor-bearing mice. NAC treatment effectively reduced the ROS levels of activated OT-I CD8^+^ T cells induced by anti-CD3 and anti-CD28 (Supplemental Figure 5A). Compared with untreated WT OT-I cells, NAC-treated WT OT-I cells were significantly more potent in promoting tumor progression (Figure 7E). In addition, treatment of WT OT-I cells with NAC reduced the frequencies of IFN-γ^+^ and Ki-67^+^ OT-I cells in tumors (Figure 7F). By contrast, NAC treatment did not alter the SUMOylation or K48-linked ubiquitination of PTEN, glycolysis, or OXPHOS in activated SENP7-deficient OT-I cells (Supplemental Figure 5, B and C, and Figure 7, C and D). Moreover, mice injected with SENP7-deficient OT-I cells treated or not with NAC displayed no apparent differences in tumor growth or the frequencies of tumor-infiltrating IFN-γ^+^ and Ki-67^+^ OT-I cells (Supplemental Figure 5, D and E). These results suggest that ROS inhibition perturbs tumor-infiltrating CD8^+^ T cell metabolism and function in an SENP7-dependent manner.

Given the potential importance of ROS in CD8^+^ T cell antitumor function, we analyzed the relationship between ROS and tumor-infiltrating CD8^+^ T cell activity in human CRC. Indeed, we found that NAC treatment restored PTEN stability in CD8^+^ T cells from CRC tissue (Figure 7G). Additionally, NAC-treated tumor-infiltrating CD8^+^ T cells exhibited decreased glycolysis and OXPHOS (Figure 7H). Accordingly, NAC treatment reduced the expression of Ki-67 in CD8^+^ T cells from CRC tissue (Figure 7I). Collectively, these results suggest that ROS are required for SENP7-dependent the metabolic fitness and antitumor function of CD8^+^ T cells.

## Discussion

In this work, we revealed a crucial role of oxidative stress in CD8^+^ T cell metabolic fitness and effector function by manipulating SENP7 activity. SENP7 was required for the metabolic reprogramming of CD8^+^ T cells, which is critical for their proliferation in vitro and antitumor immune responses in vivo. In response to TCR-induced ROS, SENP7 rapidly translocated from the nucleus to the cytoplasm to mediate PTEN deSUMOylation and in turn promoted PTEN degradation, thereby maintaining the CD8^+^ T cell metabolic state. Importantly, SENP7 sensed ROS to suppress PTEN-dependent metabolic dysfunction and functional decline in human CRC samples. These findings not only suggest that SENP7 sustains the metabolic and functional integrity of CD8^+^ T cells but also highlight what we believe to be a new link between oxidative stress and CD8^+^ T cell metabolic reprogramming.

Oxidative stress is mediated by ROS and involves CD8^+^ T cell activation, expansion, and function (18). ROS elevation upon TCR stimulation is essential for antigen-specific T cell expansion in vivo (18). Impaired oxidative metabolism with reduced ROS production results in antitumor T cell dysfunction (20). We obtained strong evidence that T cell–intrinsic ROS are indispensable for CD8^+^ T cell metabolic fitness and effector functions. Pharmacologically targeting of ROS disrupted the metabolism and impaired the antitumor functions of tumor-infiltrating CD8^+^ T cells. At a minimum, our findings suggest that SENP7 serves as a ROS sensor to facilitate CD8^+^ T cell metabolic reprogramming during naive-to-effector differentiation. Notably, aberrantly increased mitochondrial ROS in CD8^+^ T cells under hypoxia drive an exhaustion-like dysfunctional program (21). In addition, abnormal ROS accumulation induced by *G6pd* mutation disrupts CD8^+^ T cell responses to tumorigenesis (33). Physiological levels of ROS are likely critical for CD8^+^ T cell metabolic fitness and effector function, whereas excessive ROS production can cause CD8^+^ T cell dysfunction. Whether different CD8^+^ T cell subtypes have different intensities of ROS production and how SENP7 senses these different ROS levels to shape CD8^+^ T cell identity remain to be clarified.

Activation of PI3K/mTOR signaling in early-phase T cell activation involves metabolic reprogramming (9, 37, 38). PTEN acts as an inhibitor of PI3K/mTOR signaling, and its TCR signal–induced translocation to the plasma membrane is required for its phosphatase activity to terminate PI3K/mTOR signaling (34). Our previous results revealed that the lipid kinase AGK rapidly phosphorylates PTEN at Ser380, Thr382, and Thr383 during early T cell activation, thereby attenuating PTEN activity and preventing premature signal termination in CD8^+^ T cells (34). Subsequently, PTEN undergoes ubiquitination and proteasomal degradation upon TCR stimulation. Here, we provide in-depth insight into the molecular mechanism underlying PTEN degradation–mediated metabolic control in activated CD8^+^ T cells. Our current results showed that TCR-induced SENP7 cytosolic translocation facilitated the deSUMOylation, ubiquitination, and degradation of PTEN, thus allowing sustained activation of PI3K/mTOR signaling and metabolic reprogramming, thus collectively suggesting a model of SENP7-dependent PTEN restriction in activated CD8^+^ T cells.

SENP7 contains a conserved nuclear export sequence (NES) and regulates SUMO-mediated nuclear events, including transcription, DNA repair, and nuclear transport (30, 39–41). Although SENP7 has been reported to potentiate cGAS activation and contribute to the expansion of proinflammatory γδT cells in inflammatory bowel disease, the related studies used an in vivo model of SENP7 knockdown (31, 42). Using *Senp7*–conditional KO mice, we revealed a ROS-dependent function of SENP7 in regulating CD8^+^ T cell metabolic and functional states. In resting CD8^+^ T cells, SENP7 accumulated mainly in the nucleus; however, the TCR signal–induced ROS triggered the translocation of SENP7 from the nucleus to the cytoplasm, thus limiting PTEN-dependent metabolic defects. Upon treatment with the ROS inhibitor NAC or the nuclear export inhibitor LMB, TCR signal–induced SENP7 cytosolic translocation was largely blocked, resulting in PTEN accumulation in activated CD8^+^ T cells. These data suggest that TCR signals initiate SENP7 cytosolic translocation in CD8^+^ T cells in a ROS-dependent manner. However, the mechanism by which ROS cause SENP7 cytosolic translocation remains to be identified.

CD4^+^ T cells and CD8^+^ T cells have unique transcriptional and metabolic programs (43, 44). We showed here that CD4^+^ T cells had much lower expression levels of SENP7 than did CD8^+^ T cells. The difference in SENP7 expression levels in CD4^+^ T cells and CD8^+^ T cells is probably due to the different transcriptional programs. Differences in specific protein levels in CD4^+^ T cells and CD8^+^ T cells have also been reported in previous studies (34, 45). For example, the protein levels of ACAT2 are higher in CD4^+^ T cells than those in CD8^+^ T cells, whereas the protein levels of acylglycerol kinase are higher in CD8^+^ T cells than those in CD4^+^ T cells (34, 45). Therefore, the unique transcriptional program governing SENP7 expression in CD8^+^ T cells remains to be further studied.

In summary, we found that SENP7 sensed oxidative stress to sustain CD8^+^ T cell metabolic and functional fitness and was critical for effective antitumor immunity. Our results reveal an oxidative stress–sensing machinery in tumor-infiltrating CD8^+^ T cells. These findings will inform the search for oxidative stress–related agents and help to improve cancer immunotherapy outcomes.

## Methods

### Human samples.

Patient-derived PBMCs and CRC tissues were obtained from Ruijin Hospital. Solid tumor tissues were freshly isolated and digested. Immune cells were enriched by density-gradient centrifugation. Human CD4^+^ T cells and CD8^+^ T cells were purified using CD4 MicroBeads (130-045-101, Miltenyi Biotec) and CD8 MicroBeads (130-045-201, Miltenyi Biotec), respectively.

### Mice.

*Senp7*-floxed mice were generated at the GemPharmatech using a LoxP-targeting system. The *Senp7*-floxed mice were crossed with *Cd4*-Cre transgenic mice (The Jackson Laboratory) to produce age-matched *Senp7^fl/fl^* and *Senp7^fl/fl^* *Cd4*-Cre mice. The *Pten*-floxed mice (The Jackson Laboratory) were crossed with *Senp7^fl/fl^* *Cd4*-Cre mice to produce *Senp7^+/+^* *Pten^fl/fl^* *Cd4*-Cre (*Pten*-KO) and *Senp7^fl/fl^* *Pten^fl/fl^* *Cd4*-Cre (DKO) mice. *Rag1*-KO mice, OT-I TCR–transgenic mice, OT-II TCR–transgenic mice, and B6.SJL mice were from The Jackson Laboratory. Male and female mice were sex matched and used at 6 to 10 weeks of age. Mice were maintained in a specific pathogen–free (SPF) facility at the Shanghai Jiao Tong University School of Medicine.

### Cell culturing.

HEK293T cells (American Type Culture Collection [ATCC]), MC38 murine colon cancer cells (Kerafast), and MC38-OVA cancer cells (provided in-house) were cultured in DMEM medium supplemented with 10% FBS and 100 units/mL penicillin-streptomycin. B16-F10 melanoma cells (ATCC) and isolated mouse and human CD4^+^ and CD8^+^ T cells were cultured in RPMI 1640 medium supplemented with 10% FBS and 100 units/mL penicillin-streptomycin.

### Plasmids, antibodies, and reagents.

HA-tagged mouse WT or mutant (C979S) SENP7 were subcloned into the pcDNA3.1-HA vector, and Flag-tagged mouse PTEN was subcloned into the lentiviral vector pLVX-IRES-ZsGreen1. Antibodies against SENP7 (PA5-36089; 1:500 for Western blotting [WB]) were purchased from Invitrogen (Thermo Fisher Scientific). Antibodies against GAPDH (97166T; 1:1000 for WB); S6 (2217S; 1:1000 for WB); AKT (4691P; 1:1000 for WB); phosphorylated S6 (p-S6) (4858S; 1:1000 for WB); p-AKT (Thr308) (2965S; 1:1000 for WB); p-AKT (Ser473) (4060S; 1:1000 for WB); PTEN (9559S; 1:1000 for WB); SUMO2/3 (4971S; 1:1000 for WB); APC-conjugated p-S6 (D57.2.2E); and K48-ubiquitin (12805S; 1:1000 for WB) were purchased from Cell Signaling Technology. HRP-conjugated anti-HA antibody (3F10; 1:2000 for WB) was purchased from Roche. Anti-Flag (M2, F3165; 1:2000 for WB) and anti–β-actin (A2228-100UL; 1:5000 for WB) antibodies were from MilliporeSigma. The fluorochrome-conjugated antibodies against CD4 (GK1.5); CD8 (53-6.7); human CD8 (RPA-T8); CD44 (IM7); CD69 (H1.2F3); CD62L (MEL-14); Ki-67 (SolA15); Foxp3 (FJK-16s); IFN-γ (XMG1.2); TCRα2 (B20.1); CM-H2DCFDA (C6827); TNF-α (MP6-XT22); and granzyme B (GB11) were purchased from Thermo Fisher Scientific. NAC, LMB, *N*-ethylmaleimide (NEM), and CHX were purchased from MilliporeSigma. Anti–mouse PD-1 (clone J43) and isotype control IgG (hamster IgG) antibodies were purchased from Bio X Cell.

### Flow cytometry.

To detect the surface markers including CD44, CD69, CD4, and CD8, cells were stained in PBS containing 2% FBS with the indicated antibodies. Apoptotic cells were detected by FITC–annexin V and propidium iodide (PI) staining (BD Biosciences). The fluorogenic dye H2DCFDA was used to detect ROS production. Intracellular IFN-γ production was determined according to the manufacturer’s instructions (Thermo Fisher Scientific). Intracellular Foxp3 and Ki-67 staining was performed according to the manufacturer’s instructions (Thermo Fisher Scientific). For the intracellular staining of p-S6, stimulated T cells were fixed in Fix Buffer I (BD) for 10 minutes and then incubated in cold Perm Buffer III (BD) for 20 minutes, followed by antibody staining and flow cytometric analysis.

### T cell isolation and stimulation.

Naive CD4^+^ and CD8^+^ T cells were sorted using the MagniSort Mouse CD4 Naive T cell Enrichment Kit (8804-6824-74, Thermo Fisher Scientific) and the Naive CD8a^+^ T Cell Isolation Kit (30-096-543, Miltenyi Biotec), respectively. The sorted cells were stimulated with anti-CD3 (1 μg/mL) and anti-CD28 (1 μg/mL) antibodies in 96-well plates (1 × 10^5^ cells per well) for flow cytometric analysis, or stimulated with anti-CD3 (1 μg/mL) and anti-CD28 (1 μg/mL) antibodies in 6-well plates (5 × 10^6^ cells per well) for immunoblot analysis or metabolic analysis.

### Tumor models.

MC38 and MC38-OVA murine colon cancer cells were injected s.c. into 6- to 8-week-old mice (5 × 10^5^ cells per mouse). B16-F10 melanoma cells were injected s.c. into 6- to 8-week-old mice (5 × 10^5^ cells per mouse). To minimize individual variations, 6 to 8 age- and sex-matched mice in each group were used. For the adoptive transfer experiments, OT-I and OT-II cells (2 × 10^6^ cells per mouse) were injected i.v. into MC38-OVA tumor–bearing mice on 7 day after tumor injection. The tumor-challenged mice were monitored for tumor size (tumor area indicates tumor size). Tumor-induced lethality was defined as a tumor size reaching 225 mm^2^.

### RNA-Seq analysis.

Naive CD8^+^ T cells isolated from the spleens of WT and KO mice were stimulated with anti-CD3 (1 μg/mL) and anti-CD28 (1 μg/mL) antibodies for 8 hours in vitro. Fresh tumor-infiltrating WT and KO CD8^+^ T cells were isolated from the tumor-bearing mice injected s.c. with MC38 colon cancer cells (on day 7 after injection). Total RNA was isolated from in vitro–activated CD8^+^ T cells and in vivo tumor-infiltrating CD8^+^ T cells using TRIzol (Invitrogen, Thermo Fisher Scientific) and subjected to RNA-Seq using the Illumina NextSeq 500 System. The RNA-Seq data reported here are deposited in the NCBI’s Sequence Read Archive (SRA) under BioProject accession numbers PRJNA745501 and PRJNA745514.

### Metabolic measurement.

Seahorse XFe96 Extracellular Flux Analyzer (Agilent Technologies) was used for metabolic analysis. Naive CD8^+^ T cells stimulated with anti-CD3 (1 μg/mL) and anti-CD28 (1 μg/mL) antibodies for 8 hours or tumor-infiltrating CD8^+^ T cells from human CRC samples treated or not with NAC were seeded at a density of 2 × 10^5^ cells per well. The extracellular acidification rate (ECAR) and oxygen consumption rate (OCR) for each well were calculated, while the cells were subjected to the XF Glycolytic stress or the XF Cell Mito test using the following concentrations of injected compounds: 10 mM glucose, 2 μM oligomycin, 50 mM 2-deoxy-d-glucose (2-DG), 1 μM carbonyl cyanide 4-(trifluoromethoxy)phenylhydrazone (FCCP), and 0.5 μM rotenone/antimycin A. The XF Glycolytic stress or the XF Cell Mito test kits were purchased from Agilent Technologies.

### Immunoblotting, immunoprecipitation, and ELISA.

T cells were washed with ice-cold PBS and lysed on ice for 30 minutes in RIPA buffer containing protease inhibitor. Cell lysates were immunoprecipitated with the appropriate antibodies using protein A/G agarose beads. Samples were then used for immunoblot analysis with the indicated antibodies. PI3K activity was detected using the PI3-Kinase Activity ELISA Kit (K-1000s, Echelon) according to the manufacturer’s instructions.

### Statistics.

Statistical analysis was performed using GraphPad Prism (GraphPad Software). Two-way ANOVA, where applicable, was performed to compare continuous outcomes across multiple experimental groups. One-way ANOVA and 2-tailed, unpaired Student’s *t* tests were also performed. Survival curves were analyzed by log-rank (Mantel-Cox) test. A *P* value of less than 0.05 was considered statistically significant.

### Study approval.

The patient-derived PBMC and human colorectal cancer sample collection was approved by the Clinical Research Ethics Committee of Ruijin Hospital and complied with all relevant ethics regulations. Informed consent was obtained from each patient, and the study protocol was approved by the Clinical Research Ethics Committee of Ruijin Hospital and complied with all relevant ethics regulations. All animal procedures were performed according to protocols approved by the IACUC of the Shanghai Jiao Tong University School of Medicine.

## Author contributions

Z. Wu performed the experiments, analyzed the data, and wrote the manuscript. HH, QH, ZH, XLT, RD, and YY helped with mouse experiments and flow cytometry and analyzed the data. XY, RZ, Z. Wang, and QZ designed the experiments, interpreted the results, wrote the manuscript, and oversaw the research project.

## Supplementary Material

Supplemental data

## Version 1 02/10/2022

In-Press Preview

## Version 2 04/01/2022

Electronic publication
