# Supplementary material for: SENP7 senses oxidative stress to sustain metabolic fitness and antitumor functions of CD8+ T cells
Source: J Clin Invest. 2022 Apr 1;132(7):e155224. doi: 10.1172/JCI155224 (PMC8970670; doi:10.1172/JCI155224)
Supplement: Supplemental data [file jci-132-155224-s032.pdf]

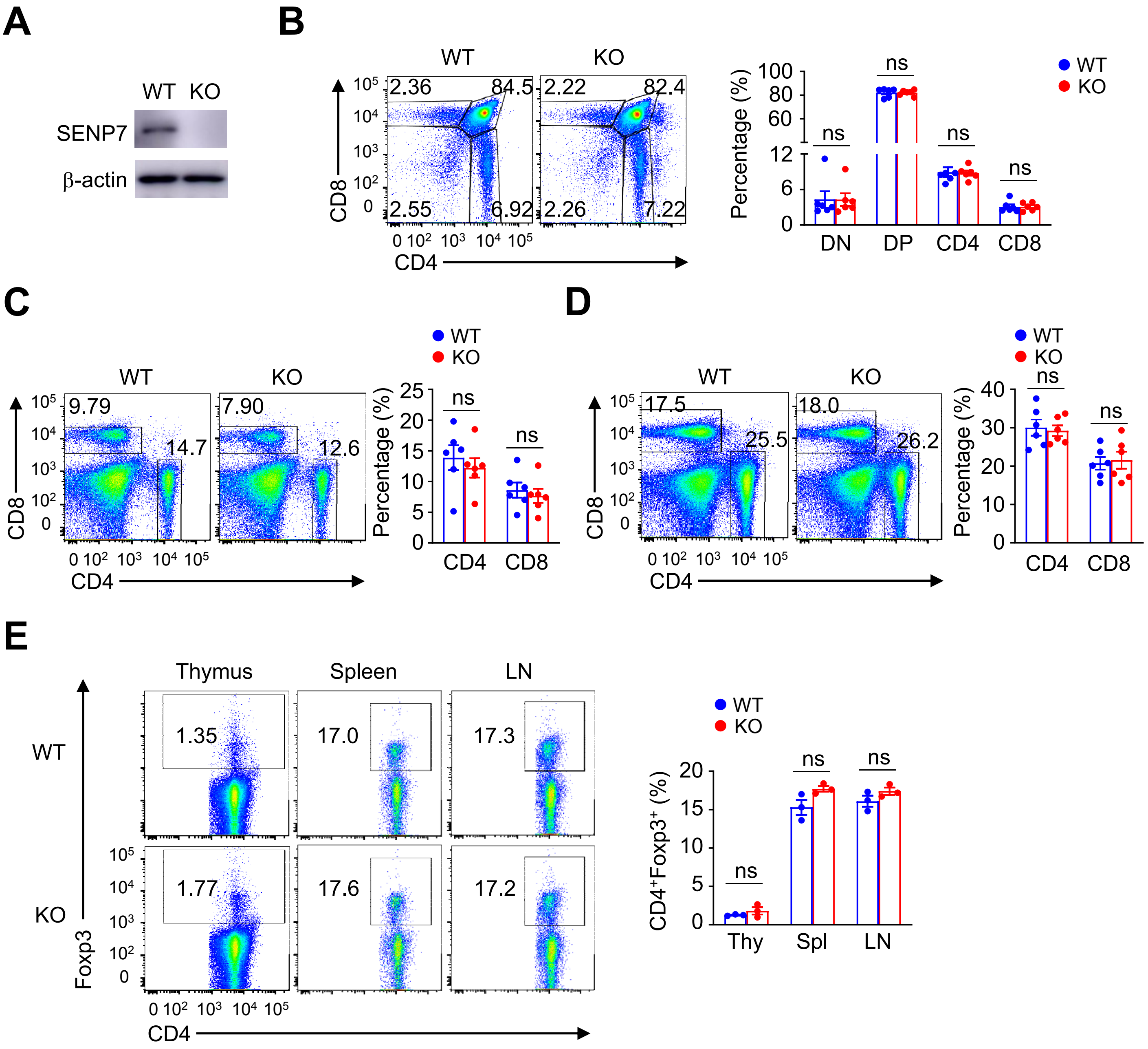

Supplementary Figure 1. T cell development in *Senp7*$^{fl/fl}$*Cd4*-Cre mice. (**A**) *Senp7*$^{fl/fl}$ mice were crossed with *Cd4*-Cre mice to obtain *Senp7*$^{fl/fl}$ (designated wild-type (WT)) and *Senp7*$^{fl/fl}$*Cd4*-Cre (designated knockout (KO)) mice. Immunoblot analysis of SENP7 in splenic CD8$^+$ T cells from WT and KO mice, showing T cell-specific SENP7 ablation. (**B**) Flow cytometric analysis of the percentage of CD4$^-$CD8$^-$ (DN), CD4$^+$CD8$^+$ (DP), CD4$^+$CD8$^-$ (CD4), CD4$^-$CD8$^+$ (CD8) cells from the thymus of 6-week-old WT and KO mice (n=5). (**C** and **D**) Flow cytometric analysis of the percentage of CD4$^+$ and CD8$^+$ T cells from the spleen (**C**) and lymph nodes (**D**) of 6-week-old WT and KO mice (n=6). (**E**) Flow cytometric analysis of the percentage of CD4$^+$Foxp3$^+$ T cells in the thymus (Thy), spleen (Spl) and lymph node (LN) of 6-week-old WT and KO mice (n=3). Data are representative of three independent experiments. ns, not statistically significant; Student's *t* test.

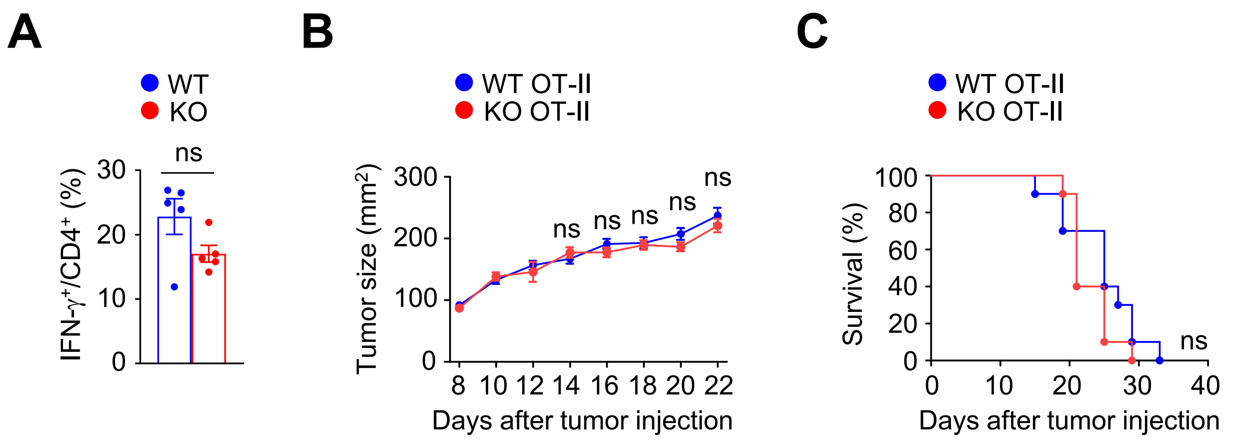

Supplementary Figure 2. Role of SENP7 in CD4⁺ T cell antitumor activity. (**A**) Flow cytometric analysis of IFN-γ-producing CD4⁺ T cells in tumors of WT and KO mice injected s.c. with MC38 tumor cells (day 14, n=5). (**B** and **C**) Tumor growth and survival curves of B6.SJL mice injected s.c. with MC38-OVA cancer cells adoptively transferred with WT OT-II or *Senp7*$^{fl/fl}$*Cd4*-Cre (KO) OT-II CD4⁺ T cells on day 7 after tumor cell inoculation (n=10). Data are representative of three independent experiments. ns, not statistically significant. (**A**) and (**B**) were analyzed by two-tailed Student's *t* test; (**C**) was analyzed by log-rank (Mantel-Cox) test.

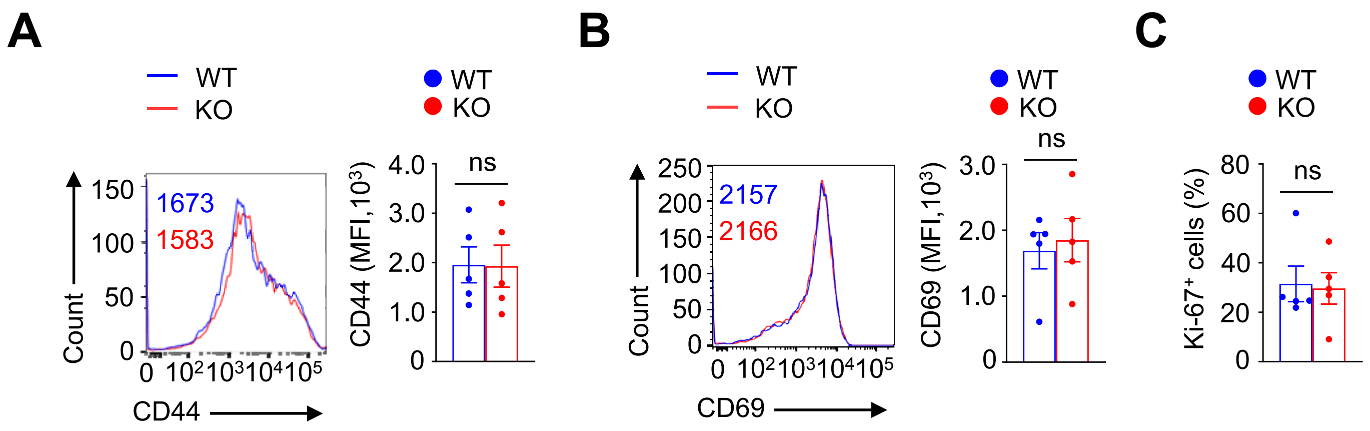

Supplementary Figure 3. SENP7 is not required for CD8⁺ T cell activation or CD4⁺ T cell proliferation. (**A** and **B**) Flow cytometric analysis of CD44 (**A**) and CD69 (**B**) expression in WT and KO CD8⁺ T cells stimulated with anti-CD3 and anti-CD28 antibodies for 1 day (n=5). (**C**) Flow cytometric analysis of the frequency of Ki-67-positive WT and KO CD4⁺ T cells stimulated with anti-CD3 and anti-CD28 antibodies for 2 days (n=5). Data are representative of three independent experiments and are presented as means ± SEM. ns, not statistically significant; Student's *t* test.

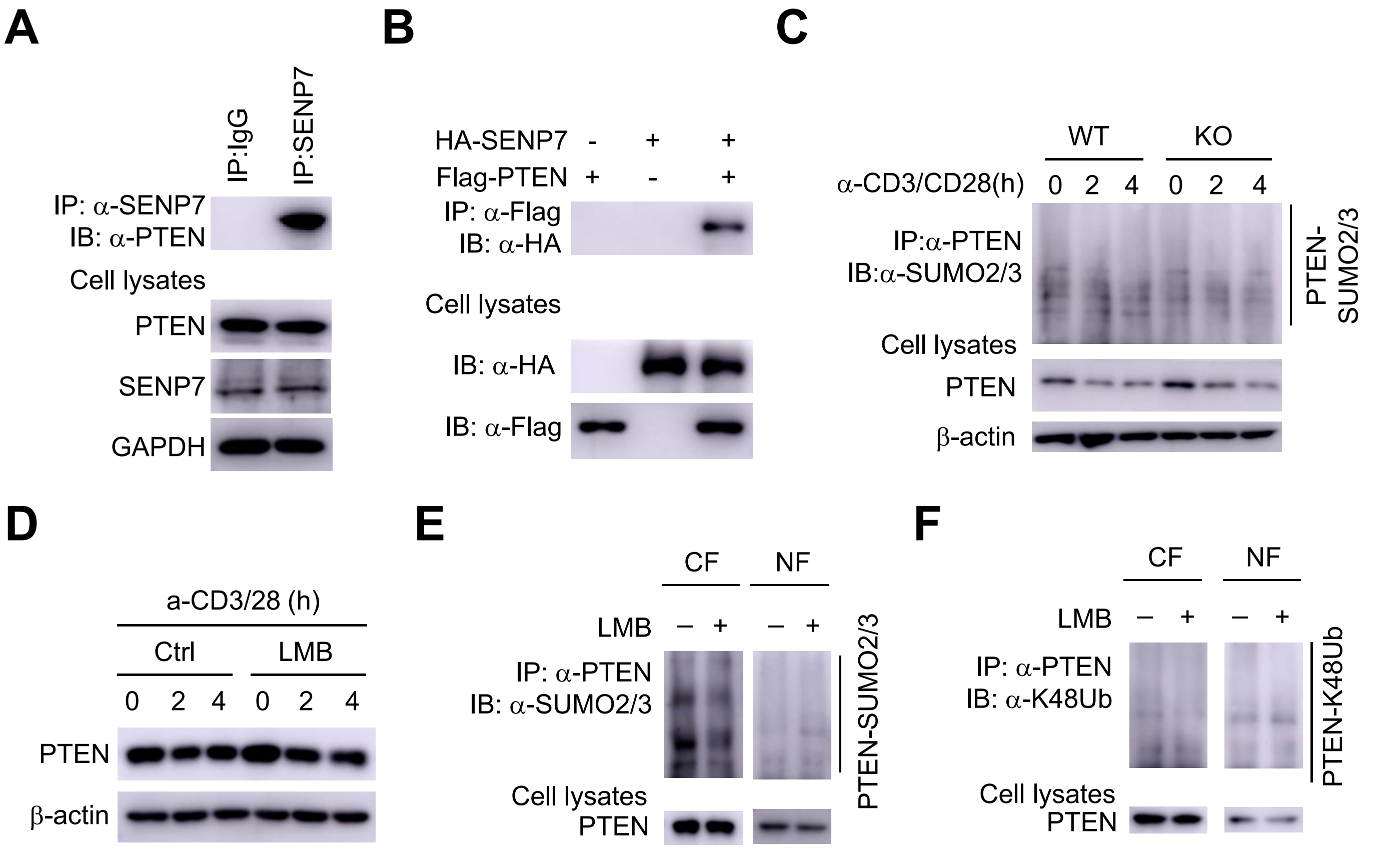

Supplementary Figure 4. PTEN activity in T cells. (**A**) WT CD8$^+$ T cells stimulated with anti-CD3 and anti-CD28 antibodies for 1 hour were immunoprecipitated with anti-SENP7 and assessed by IB with anti-PTEN. (**B**) HEK293T cells co-transfected with Flag-tagged PTEN together with HA-tagged SENP7 were immunoprecipitated with anti-Flag and assessed by IB with anti-HA. (**C**) PTEN SUMOylation assays performed by immunoprecipitating PTEN under denaturing conditions and then detecting the SUMOylated PTEN using anti-SUMO2/3 antibody in CD4$^+$ T cells stimulated with anti-CD3 and anti-CD28. (**D**) Immunoblot analysis of the indicated proteins in SENP7-deficient CD8$^+$ T cells stimulated with anti-CD3 and anti-CD28 plus LMB for 0, 2 and 4 hours. (**E** and **F**) PTEN SUMOylation (**E**) and ubiquitination (**F**) assays using nuclear (NF) and cytoplasmic (CF) fractions of SENP7-deficient CD8$^+$ T cells stimulated with anti-CD3 and anti-CD28 antibodies plus LMB for 2 hours. Data are representative of three independent experiments.

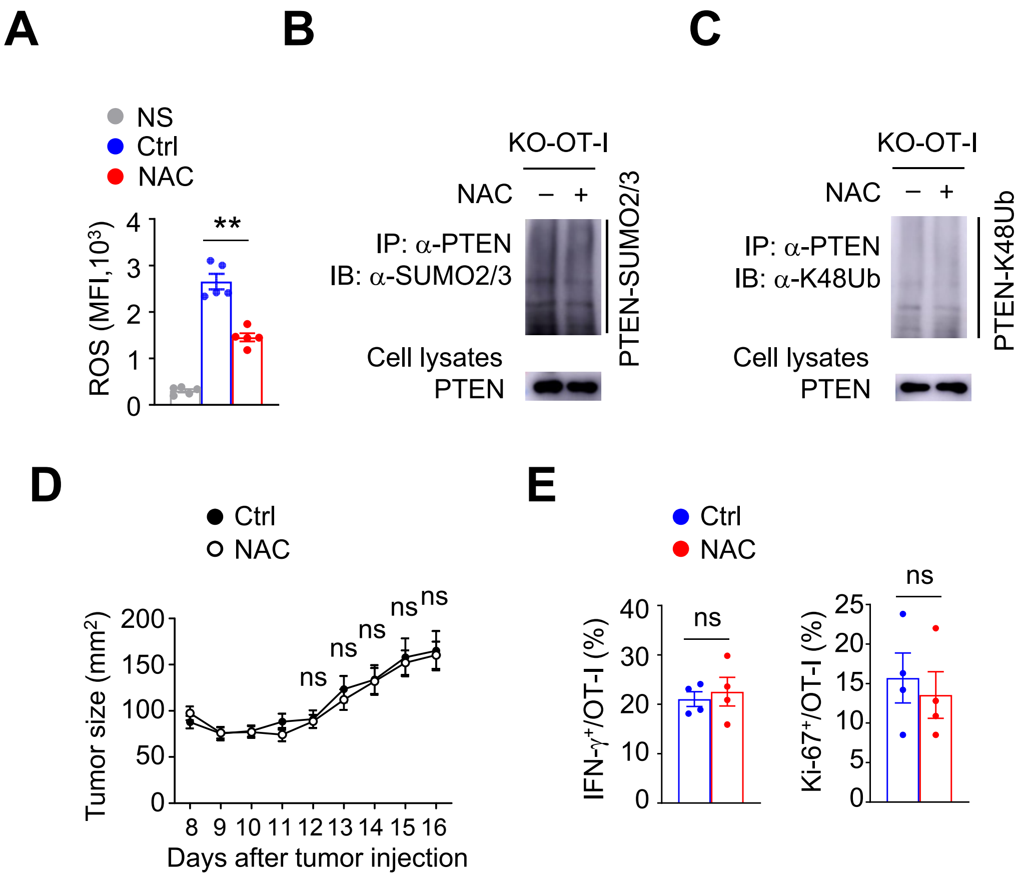

Supplementary Figure 5. Role of ROS in WT and SENP7-deficient OT-I cells. (**A**) Quantification of MFI of ROS production in wild-type OT-I cells stimulated with anti-CD3 and anti-CD28 plus 10 mM NAC for 8 hours in vitro (n=5). NS, non-stimulation with anti-CD3 and anti-CD28; Ctrl, stimulation with anti-CD3 and anti-CD28; NAC, stimulation with anti-CD3 and anti-CD28 plus NAC treatment. (**B** and **C**) PTEN SUMOylation (**B**) and ubiquitination (**C**) assays using SENP7-deficient OT-I cells stimulated with anti-CD3 and anti-CD28 antibodies plus NAC for 2 hours. (**D**) Tumor growth of MC38-OVA tumor-bearing WT mice (day 6 after tumor cell inoculation) injected intravenously with SENP7-deficient OT-I cells stimulated with anti-CD3 and anti-CD28 plus 10 mM NAC for 8 hours in vitro (n=8). (**E**) Flow cytometric analysis of the frequency of IFN-γ-producing and Ki-67-positive SENP7-deficient OT-I cells in the tumors of mice from (**D**) (day 17 after tumor injection) (n=4). Data are representative of three independent experiments and are presented as means ± SEM. The *P* value in (**A**) was determined by 1-way ANOVA with Tukey's multiple comparisons test. Student's *t* test was used in (**D**) and (**E**). ns, not statistically significant. $^{**}P < 0.01$.
